# Diffracted X-ray Blinking Tracks Single Protein Motions

**DOI:** 10.1038/s41598-018-35468-3

**Published:** 2018-11-30

**Authors:** Hiroshi Sekiguchi, Masahiro Kuramochi, Keigo Ikezaki, Yu Okamura, Kazuki Yoshimura, Ken Matsubara, Jae-Won Chang, Noboru Ohta, Tai Kubo, Kazuhiro Mio, Yoshio Suzuki, Leonard M. G. Chavas, Yuji C. Sasaki

**Affiliations:** 1 Research & Utilization Div., Japan Synchrotron Radiation Research Institute, 1-1-1, Kouto, Sayo-cho, Sayo-gun, Hyogo 567-5198 Japan; 2 Graduate School of Frontier Sciences, The University of Tokyo, 5-1-5 Kashiwanoha, Kashiwa, Chiba 277-8561 Japan; 3 Molecular Profiling Research Center for Drug Discovery, National Institute of Advanced Industrial Science and Technology, 2-4-7 Aomi, Koto-ku, Tokyo 135-0064 Japan; 4 JapanAIST-UTokyo Advanced Operando Measurement Technology Open Innovation Laboratory, 5-1-5 Kashiwanoha, Kashiwa, Chiba 277-8561 Japan; 5 Proxima-I, Synchrotron SOLEIL, L’Orme des Merisiers Saint-Aubin, BP 48 91192 Gif-sur-Yvette Cedex, France

## Abstract

Single molecule dynamics studies have begun to use quantum probes. Single particle analysis using cryo-transmission electron microscopy has dramatically improved the resolution when studying protein structures and is shifting towards molecular motion observations. X-ray free-electron lasers are also being explored as routes for determining single molecule structures of biological entities. Here, we propose a new X-ray single molecule technology that allows observation of molecular internal motion over long time scales, ranging from milliseconds up to 10^3^ seconds. Our method uses both low-dose monochromatic X-rays and nanocrystal labelling technology. During monochromatic X-ray diffraction experiments, the intensity of X-ray diffraction from moving single nanocrystals appears to blink because of Brownian motion in aqueous solutions. X-ray diffraction spots from moving nanocrystals were observed to cycle in and out of the Bragg condition. Consequently, the internal motions of a protein molecule labelled with nanocrystals could be extracted from the time trajectory using this diffracted X-ray blinking (DXB) approach. Finally, we succeeded in distinguishing the degree of fluctuation motions of an individual acetylcholine-binding protein (AChBP) interacting with acetylcholine (ACh) using a laboratory X-ray source.

## Introduction

Tracking a single molecule motion on its functional timescale has been among the aims of life sciences, and such single molecule techniques have begun to use quantum probes. Single particle analysis using cryo-transmission electron microscopy has dramatically improved the resolution in studying protein structures^1,2^ and is already shifting towards molecular motion observation^3^. X-ray free electron lasers are also being explored as routes for determining the single molecule structures of biological entities^4^. In 2000, we proposed a method to observe the intramolecular motions of individual single protein molecules by labelling them with gold nanocrystals and observing the movements of X-ray spots diffracted therefrom. This diffracted X-ray tracking^5–7^ (DXT) method can trace rotational motions within single protein molecules using X-rays with a wide energy bandwidth. Among various applications, DXT has been utilised to monitor the dynamic twisting motions of membrane proteins^8,9^ chaperonin proteins^10,11^ or other globular macromolecules^12–14^. This technique is precise to a sub-mrad angular scale.

**Figure 1:**
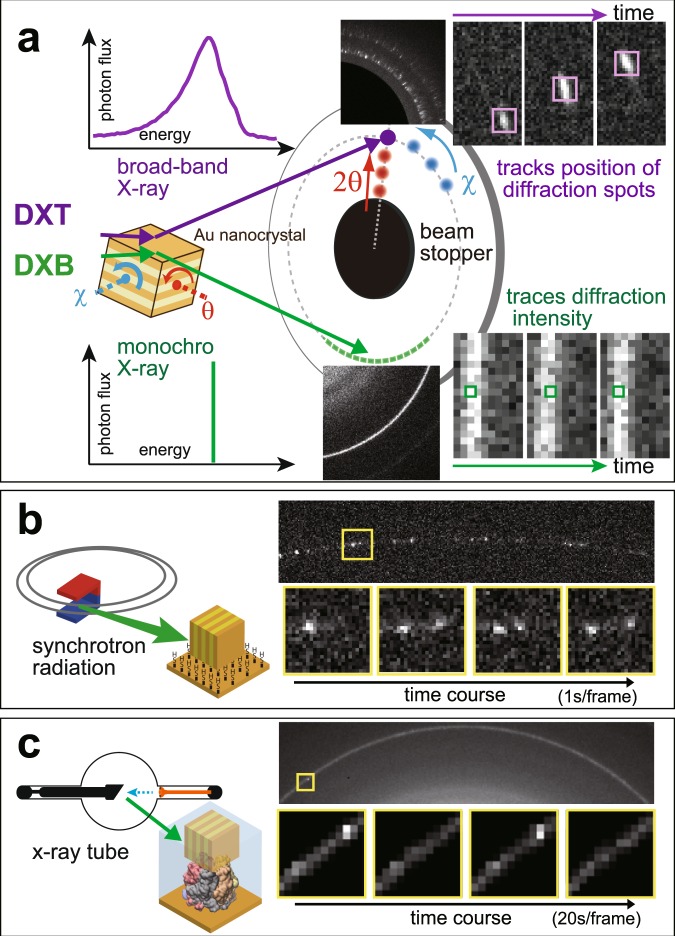
Diffracted X-ray blinking method. (**a**) Schematic representation of the classical DXT (upper) and diffracted X-ray blinking (DXB) methods (bottom). (**b**) DXB with a monochromatic X-ray from a synchrotron-radiation facility for monitoring the temperature-dependent motion of gold nanocrystals immobilised on a polyimide-substrate surface. The X-ray-diffraction intensities of the pixels at the Au(111) positions (inset of the figure) were treated with auto-correlation functions, and the decay constant was discussed in the context of DXB. (**c**) DXB with a laboratory X-ray source (Lab-DXB). Gold nanocrystals were labelled on AChBP molecules, which were immobilised on the polyimide-substrate surface. The motions of the AChBP molecules were investigated in the presence and absence of ACh in the experimental solution.

In its original description, the DXT method (Fig. 1a-upper) used a white- or pink-beam X-ray source with a wide energy bandwidth to track X-ray diffraction spots from single gold nanocrystals. There is limited availability of stable, bright white or pink hard X-ray end-stations, existing only on a few beam lines at synchrotron radiation (SR) facilities. Additionally, such X-rays physically and chemically influence the targeted biological sample via strong radiation damage. Therefore, observational times for single molecules are limited to 10 ms^9^.

Here, we apply a monochromatic X-ray source to record DXT measurements (Fig. 1a-bottom), resulting in a new technique termed diffracted X-ray blinking (DXB, Fig. 1b,c, Movies S1 and S2). The blinking is assumed to arise from discontinuous Bragg diffraction from crystals due to the motion of individual nanocrystals. Applying this property, we can analyse the blinking time trajectories of the X-ray-diffraction intensity from nanocrystals (Movie S3). The observed time trajectory was processed using the auto-correlation function (ACF), and the decay constant of the obtained ACF was correlated with the angular velocity of the gold nanocrystals. Because the nanocrystals immobilised on the substrate undergo Brownian motions in aqueous solutions^5,6^, the DXB signals from the labelled gold nanocrystals can be observed (Figs 1b and S1a). This blinking phenomenon can also be detected using a laboratory X-ray source at a quality as high as that of SR sources (Figs 1c and S1b).

We thus succeeded in monitoring the enhanced motions of acetylcholine binding protein (AChBP) interacting with acetylcholine (ACh) using the DXB approach, a laboratory X-ray source (flux: 10^8^ photons/sec.), and a typical photon-counting detector (Pilatus 100k, Dectris). The laboratory DXB (Lab-DXB) minimises the sample damage caused by incident X-rays, as the dose is less than 1/500,000th of that using an SR source (Table S1). Lab-DXB can thus be used to monitor the nanoscopic dynamics of individual proteins at longer time scales. Moreover, DXB using SR sources enabled *in vivo* observation of nanosecond high-speed X-rays from single molecules with small nanocrystal markers.

## Results and Discussion

**Figure 2:**
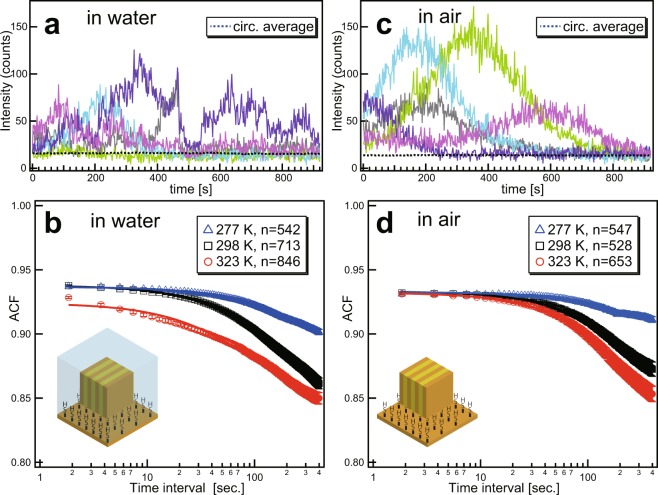
Temperature-dependent motion of Au nanocrystal immobilised on polyimide surface under water and air conditions. Intensity profiles of Au(111) positions at 298 K under water (**a**) and air (**c**) conditions. The auto-correlation function of the diffraction intensities was plotted as a function of the time interval. Open circles coloured in red, open squares coloured in black and open triangles coloured in blue in water (**b**) and in air (**d**) indicate experimental-temperature conditions of 323, 298 and 277 K, respectively. The number of ACF profiles on the Au(111) pixel used to obtain the averaged ACF profile under each temperature condition shown in (**b**) and (**d**) is shown in legends. The solid lines correspond to single exponential fitted curves, and error bars indicate standard error of ACF profiles.

An SR-source monochromatic DXB method was applied to monitor temperature-dependent motions of gold nanocrystals (Fig. 2) immobilised on polyimide substrates via a chemical-cross-linking technique (Fig. S2-upper). The nanocrystals produced ranged in diameter from 40 to 80 nm (Fig. S3). Temperature-dependent motions were investigated via ACFs of the X-ray diffraction intensities from gold nanocrystals (Au 111) immersed in water (Movie S3) or air. The blinking of the X-ray diffraction signal arose from changes in the diffraction yield (the number of photons per particle per second) of the nanocrystals in the open-probe volume, defined by the X-ray’s focal volume. Although the temperature conditions were changed during the experiments, the number of labelled nanocrystals under the focal volume of the X-ray beam remained constant. Because ordinary DXT is a transmission-type X-ray diffraction arrangement, most of the movement of the detected diffraction points was in the (θ) direction^13^. The results obtained via ACFs were treated for each pixel around the Au(111) diffraction line, except for the detector-intermodular spaces. It was evident that a temperature increase in the aqueous solution was paralleled by a sharp decrease in the ACF. Differences in the motion speeds of the diffraction points could thus be clarified from the time course of the diffracted photon signals.

The X-ray diffraction intensity increases and decreases were recorded to obtain ACF curves for each pixel on the Au(111) diffraction line, and the ACFs were fitted to an exponential decay equation as follows:

$$ACF(t)=k+A\,\exp (\,-\,Tt),$$

where ACF represents the auto-correlation function of the diffraction intensities; *k* is a constant; *A* is the functional amplitude; *Τ* is the decay constant; and *t* is the time interval. The ACF curves with a fitting parameter of *k* > 0, *A* > 0 and *T* > 0 were selected to obtain an averaged ACF under each temperature condition (Fig. 2b for the water condition, and Fig. 2d for the dry condition). The averaged ACF curves were fitted using a weighted least squares method, and the standard error of the ACFs on the Au(111) pixels were used for the weight values for fitting. The fitting parameters under each temperature are shown in Table S2. The Brownian motions of the gold nanocrystals in water were governed by the surrounding temperature and reflected in the X-ray-diffraction-intensity blinking. The decay constants of the diffracted X-ray intensity ACFs for different temperatures confirm this temperature dependence (Tables S2 and S3). During the experiments in aqueous solution (Fig. 2a,b), the decay constant increased under a high temperature condition, as shown in Table S2. During experiments under dry conditions (air; Fig. 2c,d), the decay constant became smaller than that for the solution, and the temperature correlation became complicated. The ACF of the intensity trajectory at 277 K in air remained at a constant value of approximately 1 (Fig. 2d). This result indicates that blinking was not observed under dry conditions at low temperatures, when the gold nanocrystals do not move on the substrate surface.

**Figure 3:**
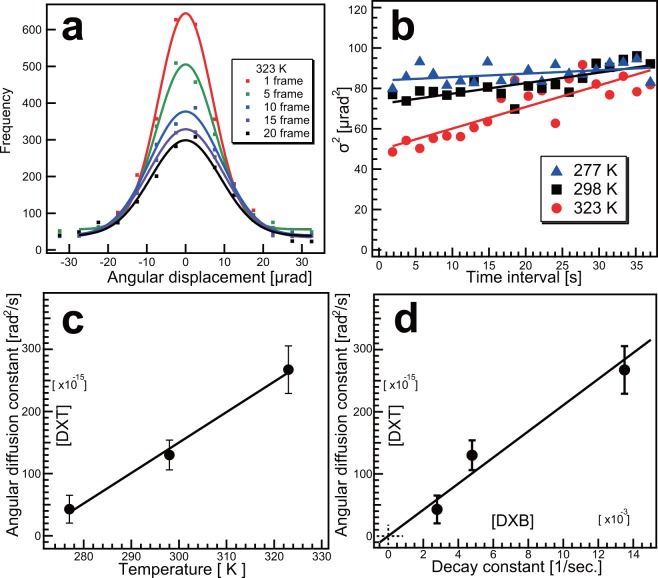
Comparison of auto-correlation analysis and diffraction spot tracking analysis. (**a**) Angular-displacement distribution of gold nanocrystals immobilised on a substrate surface in water at 323 K. (**b**) Temperature dependence of the MSD curve for gold nanocrystals on the substrate surface in water. The lines were fitted with least-squares fitting using the following equation: *MSD* = *4Dt*, where *MSD* is the mean-square angular displacement, *D* is the angular-diffusion constant and *t* is the time interval. (**c**) The relationship between the angular-diffusion constant and temperature. Solid line indicates linear regression line. (**d**) The relationship between the decay constant derived using ACF analysis (Fig. 2) and the angular-diffusion constant obtained using MSD analysis. Solid line indicates zero-intercept linear regression line. Error bars in (**c**,**d**) indicate standard deviation of angular diffusion constant.

As shown in Fig. 3, ACF analysis was compared to DXT analysis to confirm that dynamic information can be extracted from DXB. In classical DXT analysis, the positions of the diffraction spots are tracked and their angular motions analysed (Movie S2). For the present monochromatic-DXT experiments, the θ direction was represented only by a width of 3 pixels in the 2D-photon-counting detector used, yet tracking remained possible. Figure 3a shows the angular-displacement distribution of the gold nanocrystals immobilised on a substrate surface. The angular displacements for each time interval were estimated, represented in distribution histograms, and fitted with a Gaussian function, for which the standard deviations were calculated at each time interval. Mean-square-displacement (MSD) curves (Fig. 3b) were derived from the standard deviation obtained (Fig. 3a). Least-square fitting was applied to the normal-diffusion equation, allowing the angular-diffusion constant at each temperature, as well as an estimate of the angular-diffusion constant from the slope of the MSD curve, to be obtained (Table S4). The angular-diffusion constant and temperature exhibited a simple proportionality relationship (Fig. 3c), and Fig. 3d shows the almost linear relationship between the angular-diffusion constant obtained from DXT-MSD analysis and the decay constant derived from DXB-ACF analysis. Slope of zero-intercept linear regression is estimated as 2.1 × 10^−11^ rad^2^ (solid line, Fig. 3d) in this case. The degree of fluctuation of gold nanocrystal could be estimated from the decay constant from DXB from this kind of calibration line (or curve).

**Figure 4:**
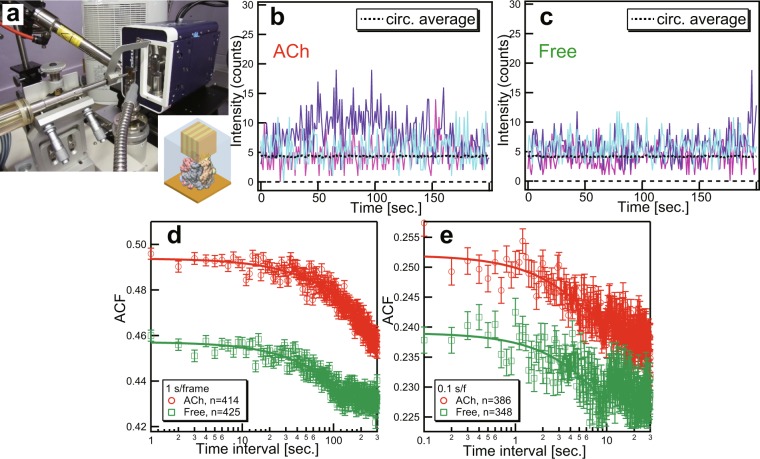
Ligand-induced motion analysis of AChBP by Lab-DXB. (**a**) Arrangement of Lab-DXB. Intensity profiles of Au(111) positions in the presence of ACh (**b**) and absence of ACh (**c**) with a time resolution of 1 sec/f. ACF analysis of AChBP at time resolutions of 1 sec/f (**d**) and 0.1 sec/f (**e**). Open circles coloured in red and open squares coloured in green were obtained in the presence and absence of acetylcholine in the experimental buffer, respectively. The number of ACF profiles used for averaging to obtain the average ACF are shown in the legends. The solid lines in (**d**,**e**) correspond to single exponential fitted curves, and error bars in (**d**,**e**) indicate standard error of ACF profiles.

To illustrate the biological implications of the Lab-DXB method (Fig. 4a), we performed motion analysis of AChBP induced by the ligand ACh at time resolutions of 1 and 0.1 sec/f (Fig. S2-bottom). During previous experiments using DXT, we already confirmed that internal motions of AChBP were enhanced by the presence of ACh within the experimental buffer^9^. The motion difference could be compared by the angular diffusion constant derived from mean angular displacement curves by DXT in the presence and absence of ACh, and those value are 8.89 × 10^−3^ and 3.78 × 10^−3^ rad^2^/sec respectively^9^. Therefore, the relative motion difference estimated by DXT is 1.53 considering the dimension of angular diffusion constant (rad^2^/sec.). To investigate the applicability of Lab-DXB, the intramolecular motions of AChBP molecules corresponding to the intensity distribution were recorded at each pixel for continuous 500-frame measurements at time intervals of 1 and 0.1 seconds. The intensity trajectories in each pixel on Au(111), as shown in Fig. 4b and c, were normalised on the basis of the total intensity trajectories from the outer pixel of Au(111) and calculated to obtain an ACF curve as described in the methods section. ACFs were fitted with one decay constant parameter (Movie S4), and these fitting parameters are shown in Table 1. In the presence of ACh in the experimental buffer, the value of the rate constant is 1.5 times larger under both measuring conditions of 1 sec/f and 0.1 sec/f in the experiments. About 1.5 motion difference between presence and absence of ACh in the experimental solution is consistent with relative motion difference obtained by DXT analysis with 0.1 ms/f^9^. These results showed that DXB analysis could qualitatively trace the enhancement of motion of AChBP in the presence of ACh within multi-time scale from sub-milliseconds to seconds scale. It would be important to evaluate the dynamic information in multi-time scales, and such experiment could be achieved by Lab-DXB and synchrotron DXB measurements. However, there are discrepancy between decay constant itself within same experimental conditions in 1 sec/f and 0.1 sec/f Lab-DXB experiments. The decay constants estimated from 1 s/f and 0.1 s/f Lab-DXB experiments showed that there are 17-fold differences between two different time scale experiments in presence and absence of ACh conditions (Table 1). We re-estimated decay constant using overlapping time points (1 sec.–30 sec.) in 1 s/f and 0.1 s/f experiments (Fig. S4 and Table S5), and the result shows that the differences of decay constants in different time scales (1 s/f and 0.1 s/f experiments) in the presence and absence of ACh are reduced to 10-fold and 5-fold, respectively (Table S5). There are still differences between decay constants from two experiments, and the mismatch might come from the difference of two experimental conditions, such as the number of gold nanocrystals on the protein in X-ray irradiation area and quality of gold nanocrystal. For a quantitative analysis in estimation of angular motion, we need a calibration curve (line) that connect decay constant obtained from DXB and actual motion of AChBP (nanocrystal) as shown in Fig. 3d.

**Table 1:** Fitting parameters and those standard errors for ACF curves in the AChBP experiments.

	*k*	*A*	*Τ*_(1/sec.)_	*chi*-*square*
1s/f ACh	(4.67 ± 0.00) × 10^−1^	(2.67 ± 0.10) × 10^−2^	(7.97 ± 0.44) × 10^−3^	175
1s/f Free	(4.13 ± 0.02) × 10^−1^	(4.40 ± 0.20) × 10^−2^	(5.49 ± 0.34) × 10^−3^	146
0.1s/f ACh	(2.36 ± 0.00) × 10^−1^	(1.61 ± 0.03) × 10^−2^	(1.41 ± 0.05) × 10^−1^	83.3
0.1s/f Free	(2.20 ± 0.01) × 10^−1^	(1.95 ± 0.06) × 10^−2^	(9.17 ± 0.50) × 10^−2^	149

Using a weighted least-squares method, ACF curves were fitted to *ACF*(*t*) = *k* + *A* exp(−*Τt*), where ACF is an auto-correlated function of diffraction intensity, *k* is a constant, *A* is the amplitude of the function, *Τ* is the decay-time constant and *t* is the time interval. The standard error of ACFs on the Au(111) pixels was used for the weight values for fitting and to obtain chi-square.

The biological molecules have different function in different time scale, for example nicotinic ACh receptors’ desensitization process ranges over a longer time-frame, such as seconds to minute time scale^15^. And long-time scale analysis also has been important in the field of molecular dynamics simulations^16^ and such analysis could be executed by DXB method with controlling the flux of incident X-ray. This is one of feature of DXB that enable to observe the molecular motion in long time scale with low X-ray dose. The internal motion of the target protein could be discussed via DXB (and DXT) on the condition that the gold nanocrystal was immobilised on the protein at a specific site and that the crystal’s motion was mainly reflected by the internal motion of the target protein. For quantitative estimation of the motion of the sample with DXB, it would be important to have homogeneous Au-crystal probes in terms of the size, crystallinity, and to evaluate the number of probes in the X-ray irradiation area. Fig. S5 shows the one of possible calibration method to estimate the sample’s angular motion quantitatively from the decay constant from DXB-ACF analysis. The sample is mount on goniometer stage, DXB measurements are executed by scanning the sample with different angular speed in tilting (θ) direction, and it evaluates the decay constant from DXB-ACF analyses with different angular scanning speed to get calibration curve that connect sample angular motion and decay constant from DXB-ACF analysis (Fig. S5). The calibration procedures were executed in each sample, and it would be almost same tendency in calibration curves unless the sample conditions are extremely different from each other, such as Au-crystal’s labelling efficiency or etc. Those Au-crystal’s labelling conditions were to be optimised considering the incident X-ray flux, the error-bars in ACF curve from 0.1 sec./f were larger compared to those obtained in 1 sec./f, and the ACF values in 1^st^ time interval of 0.1 sec./f were low (approximately 0.25) compare to those in 1 sec./f.

As previously described, DXB can be used to evaluate intramolecular motion even using a laboratory X-ray source. We found that ACF analysis using DXB could extract motion information with high sensitivity. By using monochromatic X-rays from SR sources, it is possible to evaluate internal molecular motions on a nanosecond scale with yet-smaller nanocrystal markers. By applying motion extraction from DXB to other measurement techniques, it may be possible to measure motion components via similar ACF analysis dedicated to specific methods. For example, this technique could be applied to small-angle X-ray-scattering measurements and calculation of temperature factors in X-ray protein crystallography or even in X-ray astronomy^17,18^. Moreover, it is possible to extract dynamic information with high accuracy from time-dependent signals accompanying random dynamic motion not only in the X-ray region but also in other wavelength regions. This technique could also be used to remove motion noise from still images where motion information should not be included.

In this study, we successfully used blinking X-ray diffraction to observe the internal motion of a single protein molecule using a monochromatic laboratory X-ray source for the first time. This ACF analysis is a very effective measurement, capable of acquiring dynamic information about physical or chemical phenomena over a wide range of time scales using various probes. Compared to DXT methods, DXB has a high signal-to-noise ratio and inflicts low radiation damage on the sample, using a monochromatic X-ray in DXB rather than using a pink or white X-ray beam in DXT. Therefore, DXB could be applied for more rapid measurements, such as in a sub-microsecond regime, or it could be executed using laboratory X-ray sources, as shown in this study. DXB analyses the overall Brownian motion (or fluctuation) of gold nanocrystal on the protein via the decay constant, whereas DXT can track the angular motion of gold nanocrystals in the tilting and twisting directions separately. In future DXB analysis, intensity cross-correlation analysis between the pixels on Au (111) might deduce the motion of the gold nanocrystal in more detail, though current DXB analysis uses the auto-correlation function. This approach is conceptually similar to that of X-ray photon correlation spectroscopy (XPCS)^19,20^ which records the coherency of speckle fluctuations in time and measures the timescale processes of interest. A difference from XPCS is that DXB does not require a coherent beam; however, it does require nanocrystals as markers of dynamic information.

## Methods

### DXT and DXB measurements

DXT and DXB measurements were conducted using the bending-magnet beamline BL40B2 (SPring-8, Japan). The X-ray beam was monochromatised at 0.11 nm using a double-crystal monochromator. A rhodium-coated bent-cylindrical mirror was designed for horizontal focusing at the detector position. The beam size was approximately 0.2 mm in diameter. The sample-to-detector distance was 291 mm, and time-resolved diffraction images from gold nanoparticles immobilised on the polyimide film were recorded on a 2D photon-counting detector (Pilatus 100 K, Dectris Switzerland). The exposure time per frame and interval time were, respectively, set to 1.0 sec and 1.85 sec for the gold nanocrystal measurements. The temperature of the sample was controlled by hot and cold stages (HCS302, Instec Inc). In classical DXT analysis, the motions of the spots diffracted from the gold nanocrystals on the substrate’s surface were tracked by TrackPy (v0.3.2 10.5281/zenodo.60550), and the trajectories of the diffraction spots were analysed using a custom software written within IGOR Pro (Wavemetrics, Lake Oswego, OR). Lab-DXB measurements were conducted using laboratory X-ray sources (Rigaku FR-D: Cu anode, 50 kV, 60 mA), and time-resolved diffraction images were recorded using a 2D photon-counting detector (Pilatus 100 K, Dectris, Switzerland). The sample-to-detector distance was 30 mm, and the exposure time per frame and interval time were 1.0 sec and 1.003 sec, respectively.

### Fabrication of Gold Nanocrystals

Gold nanocrystals were fabricated via epitaxial growth on cleaved KCl (100) (area: 7 × 7 mm^2^) under a 10^−4^-Pa vacuum. The shape and quality of the gold nanocrystals were confirmed using atomic-force microscopy on 1,000 particles inside 100-μm^2^ NaCl substrates.

### Analysis of Diffracted-X-ray Blinking

The intensity trajectories from each pixel around the Au(111) diffraction line, except for the intermodular rectangular area of the Pilatus detector, were extracted and analysed. The time course of the diffracted-photon signal, *I(t)*, yielded information regarding the motions of the probe particles. Fluctuations in the signal stemmed from changes in the diffraction yield (the number of photons per particle per second) of the particles in the open-probe volume defined by the focal volume of the X-ray beam. To analyse these fluctuations, the ACF of the photon intensity can be calculated as

$$ACF=\frac{\langle I(t)I(t+\tau )\rangle }{\langle I{(t)}^{2}\rangle },$$

where the angular brackets, $\langle \rangle$, indicate a time average; *I(t)* is the number of diffracted photons as a function of time; and τ is the delay time^21,22^. ACFs were treated for each pixel on the Au(111)-diffraction line, except for the detector-intermodular spaces, and fit to single exponential curves in the range during the first 10% of observed time (i.e., the fitting range was from 1 to 101 frames in the case of a 1,000-frame experiment), ACF = *k* + A exp (−*T*t), where *k* is a constant, *A* is the amplitude, and *T* is the decay constant. ACF curves with the fitting parameters of k > 0, A > 0, and T > 0 were included to estimate averaged ACF curves under each condition. The averaged ACF curves were fitted using a weighted least-squares method using weight values of the standard error for averaged ACFs. The chi-square values for fittings are shown in Tables 1, S2 and S3. During the measurements using SR, the storage ring at SPring-8 was run in top-up mode with a fixed current of 99.5 mA. In the case of Lab-DXB, the intensity trajectories were normalised on the basis of the total background intensity from the outer pixels of Au(111).

### Sample Preparation

A 50-μm-thick polyimide film (Du Pont-Toray, Tokyo, Japan) was soaked in 1-M KOH (313 K) for 15 minutes to expose carboxyl groups on the polyimide surface and washed with MilliQ water. The polyimide surface was mixed to react with 20 mg/mL of N-hydroxysuccinimide (Thermo Fisher Scientific) and 30 mg/mL of 1-ethyl-3-(3-dimethylaminopropyl)carbodiimide hydrochloride (Thermo Fisher Scientific) in a 10-mM MES buffer (pH 4.5) for 30 min at room temperature. The activated substrate surface was washed with distilled water and reacted with cysteamine to immobilise the gold nanocrystals on the substrate surface and soaked in 10-mM *N*-(5-Amino-1-carboxypentyl) iminodiacetic acid (AB-NTA, dojindo, Japan) in a phosphate buffer for 3 hr to expose NTA on the substrate surface for immobilisation of recombinant AChBP. The NTA-modified surface was soaked in a 100-mM NiSO_4_ solution (in 50 mM MOPS, pH 7.0) for 4 hr at RT. The Ni-treated substrate was rinsed with MilliQ water and reacted with a His-tagged AChBP solution (0.2 mg/mL in PBS) overnight at 277 K.

Gold nanocrystals were obtained via epitaxial growth on a NaCl or KCl (100) surface and dissolved with detergent, n-decyl-β-D-maltoside (Dojindo Laboratories), in 50-mM MOPS (pH 7.0). The gold-nanocrystal solution was applied to a cysteamine-modified surface, and AChBP was immobilised upon the substrate and incubated for 7 hr at 277 K. Recombinant AChBP molecules were expressed in *E*. *coli* and purified according to the method described in a previous report^9^. AChBP was examined in the presence or absence of 0.1 mM ACh.

## Electronic supplementary material

Supplementary Information

Video 1

Video 2

Video 3

Video 4

## Data Availability

The datasets generated during and/or analysed during the current study are available from the corresponding author on reasonable request.

## References

[1] Henderson R (1990). Model for the structure of bacteriorhodopsin based on high-resolution electron cryo-microscopy. J. Mol. Biol..

[2] Merk A (2016). Breaking Cryo-EM Resolution Barriers to Facilitate Drug Discovery. Cell.

[3] Frank Joachim (2017). Time-resolved cryo-electron microscopy: Recent progress. Journal of Structural Biology.

[4] Spence JCH, Weierstall U, Chapman HN (2012). X-ray lasers for structural and dynamic biology. Reports Prog. Phys..

[5] Sasaki Y (2000). Tracking of individual nanocrystals using diffracted x rays. Phys. Rev. E.

[6] Sasaki YC (2001). Picometer-Scale Dynamical X-Ray Imaging of Single DNA Molecules. Phys. Rev. Lett..

[7] Okumura Y, Oka T, Kataoka M, Taniguchi Y, Sasaki YC (2004). Picometer-scale dynamical observations of individual membrane proteins: The case of bacteriorhodopsin. Phys. Rev. E.

[8] Shimizu H (2008). Global twisting motion of single molecular KcsA potassium channel upon gating. Cell.

[9] Sekiguchi H (2015). Real Time Ligand-Induced Motion Mappings of AChBP and nAChR Using X-ray Single Molecule Tracking. Sci. Rep..

[10] Sekiguchi H (2013). ATP Dependent Rotational Motion of Group II Chaperonin Observed by X-ray Single Molecule Tracking. PLoS One.

[11] Yamamoto YY (2016). Characterization of group II chaperonins from an acidothermophilic archaeon Picrophilus torridus. FEBS Open Bio.

[12] Matsushita Y (2015). Time-resolved X-ray Tracking of Expansion and Compression Dynamics in Supersaturating Ion-Networks. Sci. Rep..

[13] Kozono H (2015). Single-Molecule Motions of MHC Class II Rely on Bound Peptides. Biophys. J..

[14] Matsushita Y (2017). Nanoscale Dynamics of Protein Assembly Networks in Supersaturated Solutions. Sci. Rep..

[15] Giniatullin R, Nistri A, Yakel JL (2005). Desensitization of nicotinic ACh receptors: shaping cholinergic signaling. Trends Neurosci..

[16] Klepeis JL, Lindorff-Larsen K, Dror RO, Shaw DE (2009). Long-timescale molecular dynamics simulations of protein structure and function. Curr. Opin. Struct. Biol..

[17] Cirtain JW (2007). Evidence for Alfven Waves in Solar X-ray Jets. Science (80-.)..

[18] Sterling AC, Moore RL, Falconer DA, Adams M (2015). Small-scale filament eruptions as the driver of X-ray jets in solar coronal holes. Nature.

[19] Shinohara Y, Watanabe A, Kishimoto H, Amemiya Y (2013). Combined measurement of X-ray photon correlation spectroscopy and diffracted X-ray tracking using pink beam X-rays. J. Synchrotron Radiat..

[20] Nogales A, Fluerasu A (2016). X Ray Photon Correlation Spectroscopy for the study of polymer dynamics. Eur. Polym. J..

[21] Brauer S (1995). X-ray intensity fluctuation spectroscopy observations of critical dynamics in Fe3Al. Phys. Rev. Lett..

[22] Shpyrko OG (2007). Direct measurement of antiferromagnetic domain fluctuations. Nature.

