# Supplementary material for: Diffracted X-ray Blinking Tracks Single Protein Motions
Source: Sci Rep. 2018 Nov 30;8:17090. doi: 10.1038/s41598-018-35468-3 (PMC6269541; doi:10.1038/s41598-018-35468-3)
Supplement: Supplementary file 1 — Supplementary Information [file 41598_2018_35468_MOESM1_ESM.pdf]

Supplementary information

Diffracted X-ray Blinking Tracks Single Protein Motions

Hiroshi Sekiguchi[1,*], Masahiro Kuramochi[2], Keigo Ikezaki[2], Yu Okamura[2], Kazuki Yoshimura[2], Ken Matsubara[2], Jae-won Chang[2], Noboru Ohta[1], Tai Kubo[3,4], Kazuhiro Mio[3,4], Yoshio Suzuki[2], Leonard M. G. Chavas[5], and Yuji C. Sasaki[1, 2, 4, *]
[1] Research & Utilization Div., Japan Synchrotron Radiation Research Institute, 1-1-1, Kouto, Sayo-cho, Sayo-gun, Hyogo 567-5198, Japan
[2] Graduate School of Frontier Sciences, The University of Tokyo, 5-1-5 Kashiwanoha, Kashiwa, Chiba, 277-8561, Japan
[3] Molecular Profiling Research Center for Drug Discovery, National Institute of Advanced Industrial Science and Technology, 2-4-7 Aomi, Koto-ku, Tokyo 135-0064, Japan
[4] JapanAIST-UTokyo Advanced Operando Measurement Technology Open Innovation Laboratory, 5-1-5 Kashiwanoha, Kashiwa, Chiba, 277-8561, Japan
[5] Proxima-I, Synchrotron SOLEIL, L'Orme des Merisiers Saint-Aubin, BP 48 91192 Gif-sur-Yvette Cedex France

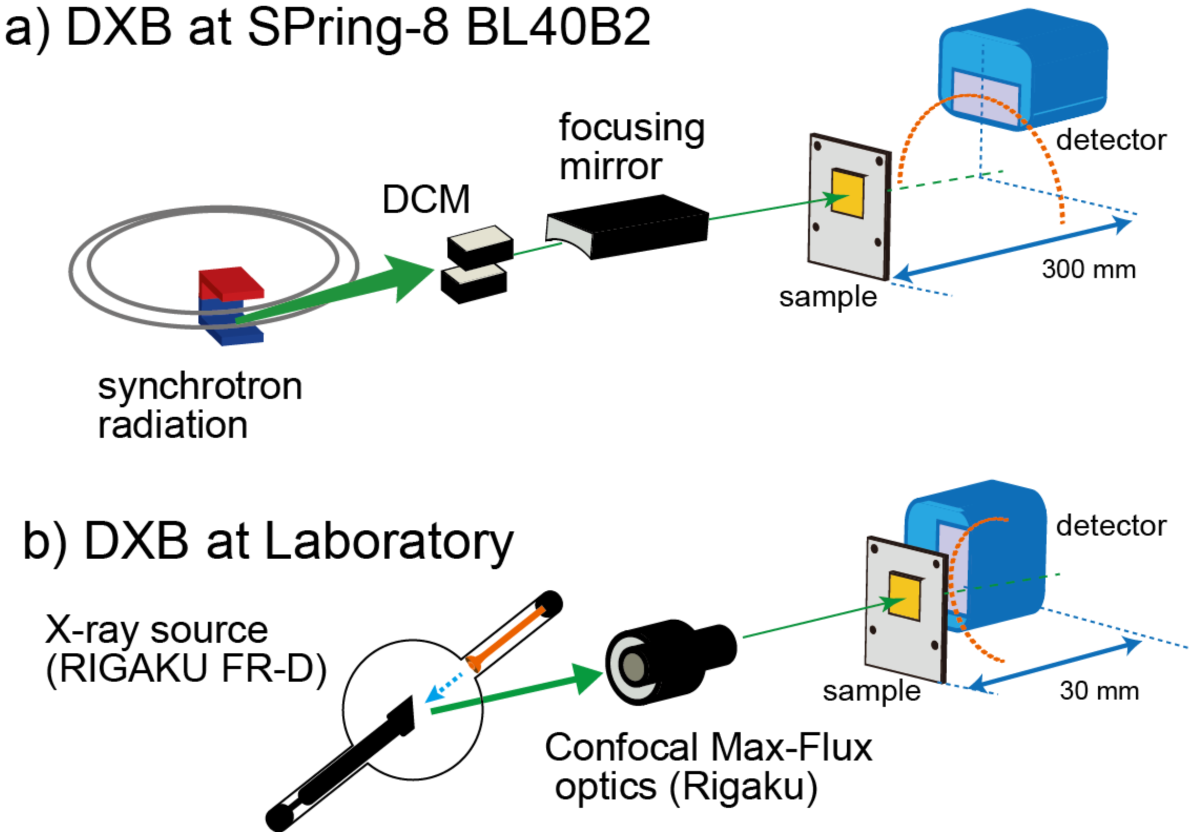

Fig. S1: Instrumentation of DXB at the synchrotron radiation facility and at the laboratory.

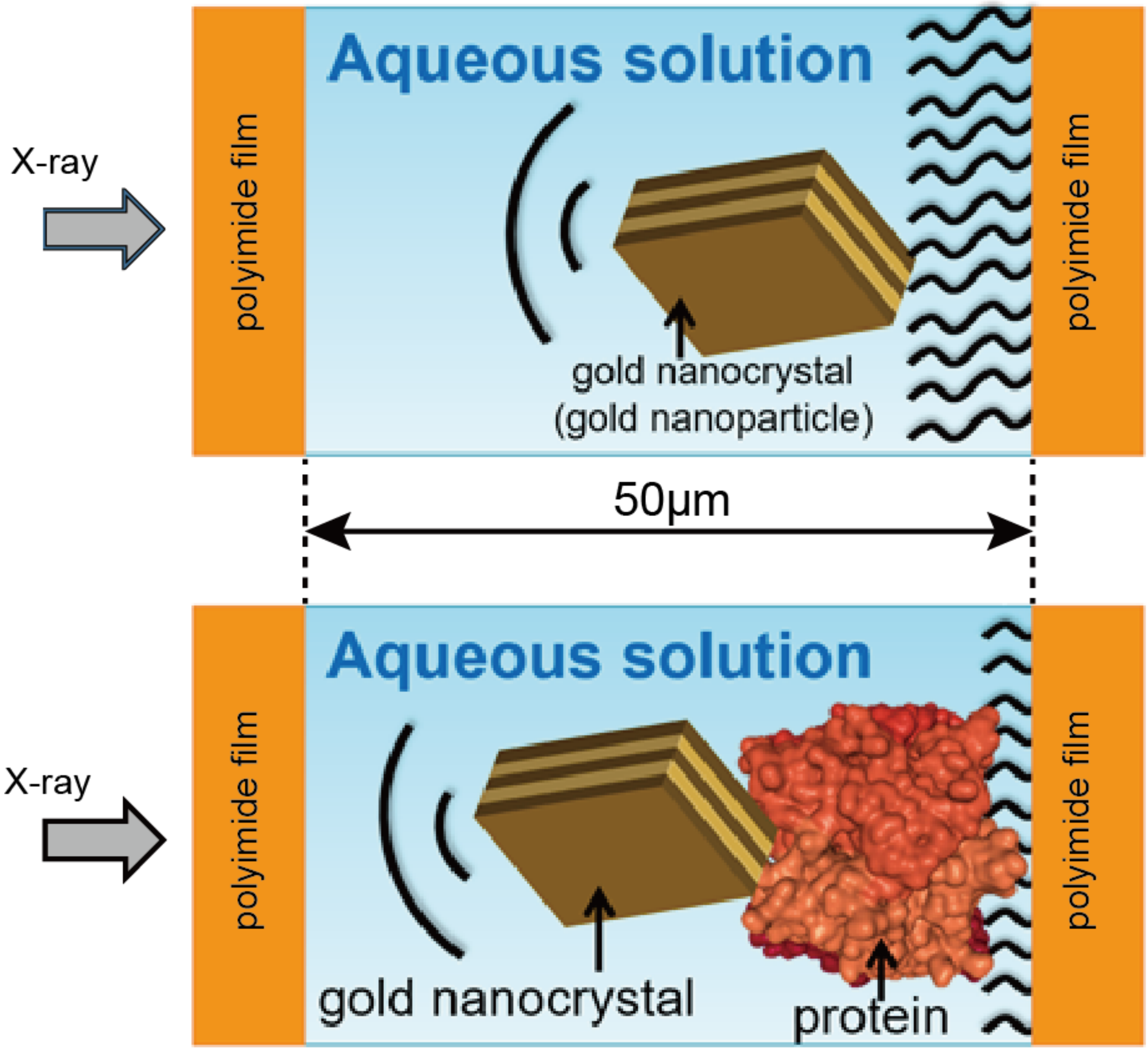

Fig. S2: Schematics of samples for DXB measurements.

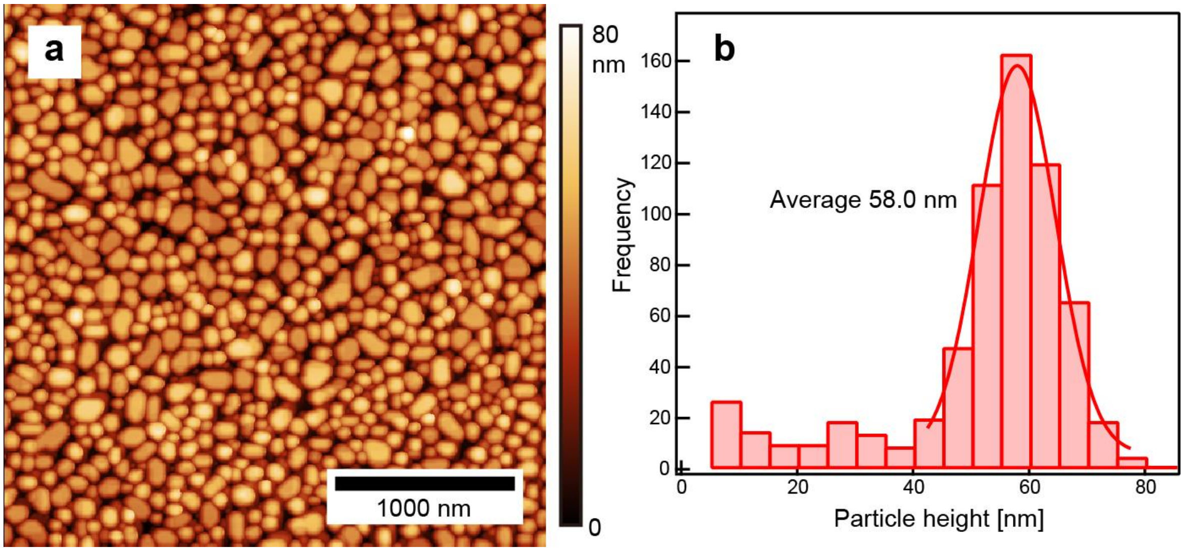

Fig. S3: Size of gold nanocrystal. AFM image of a gold nanocrystal (A) and its height distribution (B). The AFM image was obtained using an MM-AFM NanoScope IIIa (Bruker) under an air condition. The height of gold nanocrystals was analysed using the grain analysis function of the Gwyddion software[1].

1. Nečas, D. & Klapetek, P. Gwyddion: an open-source software for SPM data analysis. *Cent. Eur. J. Phys.* **10,** 181–188 (2012).

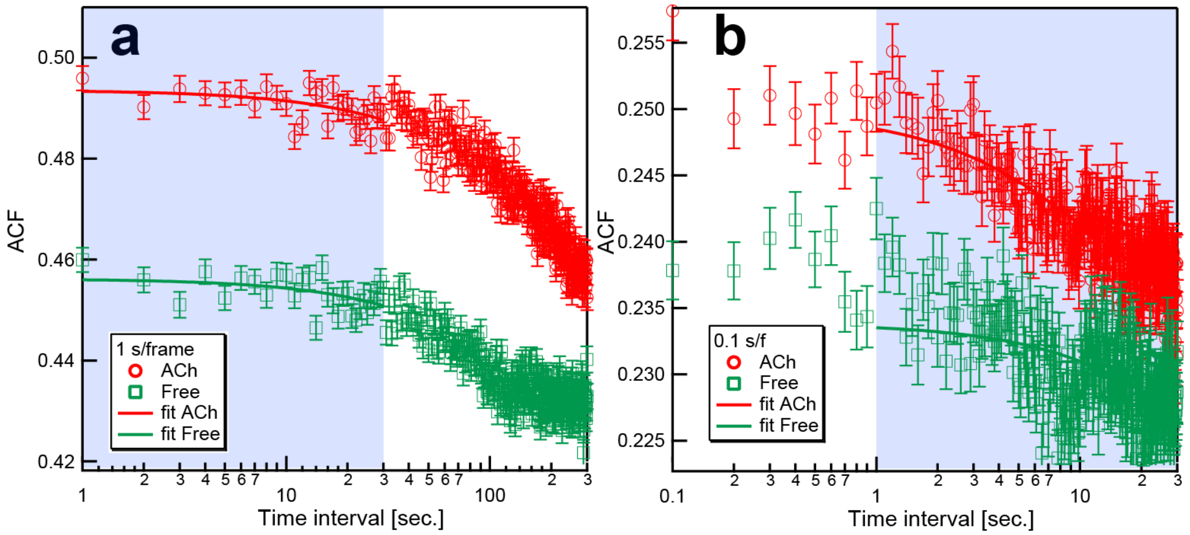

Fig. S4: Lab-DXB ACF analysis of AChBP at time resolutions of 1 sec/f (a) and 0.1 sec/f (b) with same fitting range (from 1 to 30 seconds). Fitting parameters and those standard errors for ACF curves are shown at Table S5.

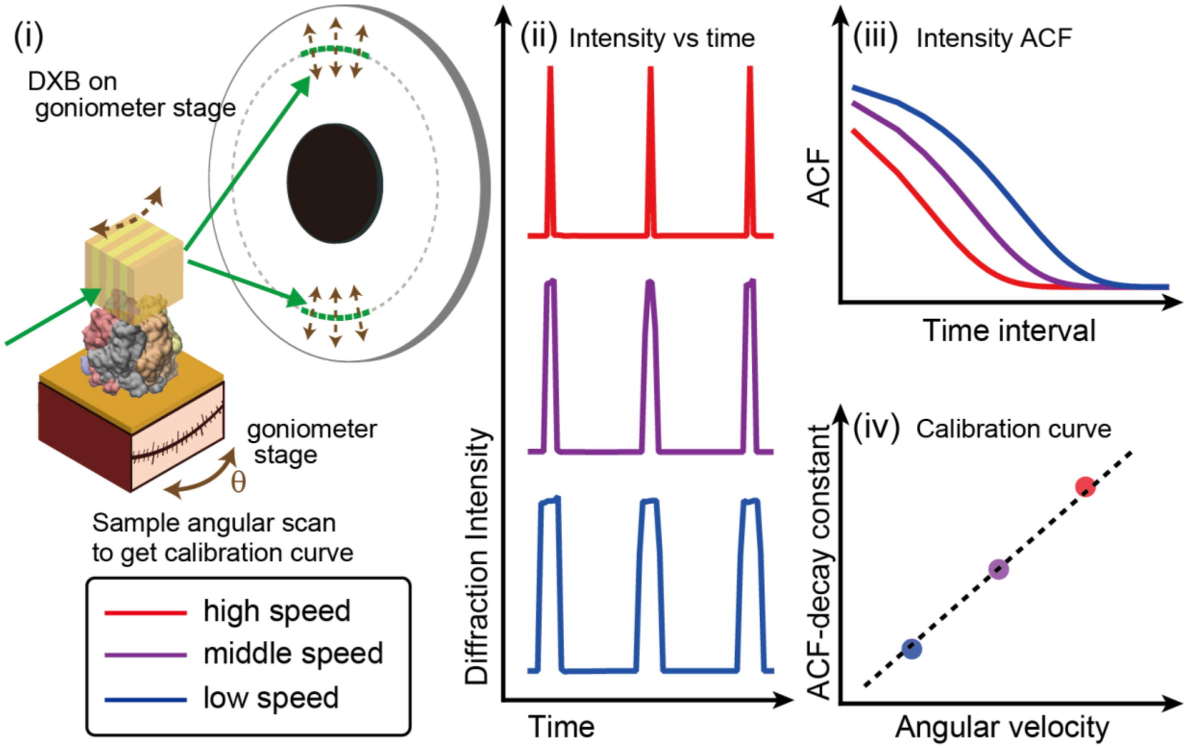

Fig. S5: Calibration method for evaluating the relationship between sample's angular motion and decay constant from DXB-ACF analysis. The calibration procedures are as follows, (i) the sample is mount on goniometer stage which scan the sample in tilting (θ) direction, (ii) DXB measurement are executed on rotation centre of goniometer stage by scanning the sample with different angular speed, and record transition of diffraction intensity from nanocrystal on sample, (iii) DXB-ACF analysis are executed to get ACF decay constant at different sample angular scanning speed, and (iv) the relationship between ACF-decay constant and sample angular scanning speed is evaluated to get calibration curve in DXB.

	X-ray source	Photon flux (photon/s)	Beam size (mm)	Energy (keV)	Dose rate (for water) Gy/s
Usual DXT (BL40XU)	Helical undulator	1.0×10^{13} (DXT mode)	0.15(H) ×0.05 (V)	15.2	390000
DXB (BL40B2)	Bending magnet	2.7×10^{10}	0.29(H) ×0.24 (V)	11.3	240
Lab-DXB (FR-D)	FR-D	4.0×10^{8}	0.8(H) ×0.8 (V)	8.0	0.8

Table S1: DXT and DXB measurements with different X-ray sources.

	k	*A*	*T* (1/sec.)	*chi-square*
Water 277 K	$(8.77 \pm 0.01) \times 10^{-1}$	$(5.96 \pm 0.11) \times 10^{-2}$	$(2.79 \pm 0.06) \times 10^{-3}$	5.32
Water 298 K	$(8.47 \pm 0.01) \times 10^{-1}$	$(9.06 \pm 0.06) \times 10^{-2}$	$(4.79 \pm 0.04) \times 10^{-3}$	4.82
Water 323 K	$(8.65 \pm 0.01) \times 10^{-1}$	$(5.86 \pm 0.01) \times 10^{-2}$	$(1.35 \pm 0.01) \times 10^{-2}$	129.4

Table S2: Fitting parameters and those standard errors for ACF curves under a water condition. ACF curves were fitted using weighted least-squares fitting to the following equation: $ACF(t) = k + A \exp(-Tt)$, where ACF is an auto-correlated function of diffraction intensity, *k* is a constant, *A* is the amplitude of the function, *T* is the decay constant and *t* is the time interval. The standard error of ACFs on the Au(111) pixels were used for the weight values for fitting and to obtain chi-square.

	k	A	T(1/sec)	*chi-square*
Air 277 K	$(8.75 \pm 0.00) \times 10^{-1}$	$(5.74 \pm 0.00) \times 10^{-2}$	$(1.64 \pm 0.00) \times 10^{-3}$	25.3
Air 298 K	$(8.14 \pm 0.00) \times 10^{-1}$	$(1.18 \pm 0.00) \times 10^{-1}$	$(2.18 \pm 0.00) \times 10^{-3}$	11.6
Air 323 K	$(7.77 \pm 0.00) \times 10^{-1}$	$(1.55 \pm 0.00) \times 10^{-1}$	$(2.43 \pm 0.00) \times 10^{-3}$	2.54

Table S3: Fitting parameters and those standard errors for ACF curves in air. ACF curves were fitted using weighted least-squares fitting to the following equation: $ACF(t) = k + A \exp(-Tt)$, where ACF is an auto-correlated function of diffraction intensity, k is a constant, A is the amplitude of the function, T is the decay constant and t is the time interval. The standard error of ACFs on the Au(111) pixels were used for the weight values for fitting and to obtain chi-square.

	a [mrad]	D [rad^2/s]
Water 277 K	9.16×10^{-3}	4.28×10^{-14}
Water 298 K	8.50×10^{-3}	1.30×10^{-13}
Water 323 K	7.03×10^{-3}	2.67×10^{-13}

Table S4: Fitting parameters for MSD curves in Figure 3B. Using a least-squares technique, the lines in Figure 3B were fitted to $MSD=a^2+ 4Dt$, where MSD is the mean-square angular displacement, a is square root of the intercept of MSD curve, D is the angular-diffusion constant and t is a time interval.

	k	*A*	$T_{(1/sec.)}$	*chi-square*
1 s/f ACh	4.67×10^{-1} [fixed]	$(2.65 \pm 0.02) \times 10^{-2}$	$(8.34 \pm 0.40) \times 10^{-3}$	37.4
1 s/f Free	4.13×10^{-1} [fixed]	$(4.30 \pm 0.02) \times 10^{-2}$	$(4.40 \pm 0.23) \times 10^{-3}$	42.0
0.1 s/f ACh	2.36×10^{-1} [fixed]	$(1.38 \pm 0.00) \times 10^{-2}$	$(9.50 \pm 0.02) \times 10^{-2}$	304
0.1 s/f Free	2.20×10^{-1} [fixed]	$(1.43 \pm 0.00) \times 10^{-2}$	$(2.22 \pm 0.09) \times 10^{-2}$	462

Table S5: Fitting parameters and those standard errors for ACF curves in the AChBP experiments with fitting time range from 1 to 30 seconds (Fig. S4). Using a weighted least-squares method, ACF curves were fitted to $ACF(t) = k + A \exp(-T\,t)$, where ACF is an auto-correlated function of diffraction intensity, *k* is a constant, *A* is the amplitude of the function, *T* is the decay-time constant and *t* is the time interval. *k* is fixed on a value obtained in Table 1. The standard error of ACFs on the Au(111) pixels was used for the weight values for fitting and to obtain chi-square.

Video S1: Concept of DXB measurement.
Video S2: Comparison of DXT and DXB measurements
Video S3: DXB measurement using synchrotron radiation.
Video S4: DXB measurement using a laboratory X-ray source
